# Health status in older hospitalized patients with cancer or non-neoplastic chronic diseases

**DOI:** 10.1186/1471-2318-5-10

**Published:** 2005-08-25

**Authors:** Andrea Corsonello, Claudio Pedone, Luciana Carosella, Francesco Corica, Bruno Mazzei, Raffaele Antonelli Incalzi, the Gruppo Italiano di Farmacovigilanza nell'Anziano (GIFA) investigators

**Affiliations:** 1 Istituto Nazionale di Ricovero e Cura per Anziani (INRCA), Cosenza, Italy; 2 Cattedra di Geriatria, Università Campus-BioMedico, Rome, Italy; 3 Centro di Medicina dell'Invecchiamento, Policlinico "Agostino Gemelli", Università Cattolica del Sacro Cuore, Rome, Italy; 4 Dipartimento di Medicina Interna, Università degli Studi di Messina, Messina, Italy

## Abstract

**Background:**

Whether cancer is more disabling than other highly prevalent chronic diseases in the elderly is not well understood, and represents the objective of the present study.

**Methods:**

We used data from the Gruppo Italiano di Farmacovigilanza nell'Anziano (GIFA) study, a large collaborative observational study based in community and university hospitals located throughout Italy. Our series consisted of three groups of patients with non-neoplastic chronic disease (congestive heart failure, CHF, N = 832; diabetes mellitus, N = 939; chronic obstructive pulmonary disease, COPD, N = 399), and three groups of patients with cancer (solid tumors without metastasis, N = 813; solid tumors with metastasis, N = 259; leukemia/lymphoma, N = 326). Functional capabilities were ascertained using the activities of daily living (ADL) scale, and categorical variables for dependency in at least 1 ADL or dependency in 3 or more ADLs were considered in the analysis. Cognitive status was evaluated by the 10-items Hodgkinson Abbreviated Mental Test (AMT).

**Results:**

Cognitive impairment was more prevalent in patients with CHF (28.0%) or COPD (25.8%) than in those with cancer (solid tumors = 22.9%; leukemia/lymphoma = 19.6%; metastatic cancer = 22.8%). Dependency in at least 1 ADL was highly prevalent in patients with metastatic cancer (31.3% vs. 24% for patients with CHF and 22.4% for those with non-metastatic solid tumors, p < 0.001). In people aged 80 years or more, metastatic cancer was not associated with increased prevalence of physical disability. In multivariable analysis, metastatic cancer was associated with a greater prevalence of physical (OR 2.09, 95%CI 1.51–2.90) but not cognitive impairment (OR 1.34, 95%CI 0.94–1.91) with respect to CHF patients. Finally, diabetes was significantly associated with cognitive impairment (OR 1.40, 95%CI 1.11–1.78).

**Conclusion:**

Cancer should not be considered as an ineluctable cause of severe cognitive and physical impairment, at least not more than other chronic conditions highly prevalent in older people, such as CHF and diabetes mellitus.

## Background

Acute disabling conditions such as stroke or hip fracture have obvious and dramatic effects on functional capabilities, whereas chronic conditions which do not cause a segmental motor deficit have a more complex and less predictable effect. Clinical observations suggest that chronic diseases may be associated with different patterns of physical decline. For example, a distinctive pattern of disability has been found in chronic obstructive pulmonary disease (COPD) compared with that characterizing patients with congestive heart failure (CHF) or diabetes mellitus [1].

Physical dependency can be seen as the end result of the complex interaction among physical, cognitive and affective factors. Cancer is commonly perceived as a highly disabling condition, whereas the impact of other chronic conditions such as diabetes mellitus or hypoxemic COPD on functional capabilities is underestimated [2,3].

Given et al reported that, at the time of the first diagnosis, older cancer patients have considerably better physical function than persons of the same age from the general population [4]. Thus, cancer, on average, might not be more disabling than other highly prevalent chronic diseases in the elderly. Clarifying this issue might be relevant to quantify the needs of care besides the expenditure directly related to the treatment of cancer as well as to select patients most likely to benefit from a comprehensive assessment program. [5]. Indeed, interventions guided by geriatric assessment have positive effects on a number of important health outcomes in frail older patients in different settings [6-9]. However, older cancer patients are underrepresented in geriatric assessment and intervention trials [10]. This makes desirable to clarify the impact of cancer on physical and mental capabilities in comparison with that of conditions such as CHF, COPD and diabetes mellitus which were highly prevalent in geriatric series proven to benefit form geriatric assessment [6-9]. This is the objective of the present study.

## Methods

We used data from the Gruppo Italiano di Farmacovigilanza nell'Anziano (GIFA) study, a large collaborative observational study that periodically surveys drug consumption, occurrence of adverse drug reactions (ADR), and quality of hospital care. We used data on patients consecutively admitted to the participating centers during the 4 months surveys carried out in 1993, 1995, 1997 and 1998. Methods of the GIFA have been previously described [11]. Briefly, after obtaining a written informed consent, all patients admitted to the 81 participating wards of Geriatric or Internal Medicine in tertiary hospitals located throughout Italy were enrolled and followed until discharge. There were no inclusion or exclusion criteria. The majority of patients were admitted from the Emergency Room at each hospital, and the diagnosis made by the on-call physician in the Emergency Room was recorded. For each patient a questionnaire was completed at admission and updated daily by a study physician who received specific training for the study.

Data recorded included demographic characteristics, drugs taken prior to admission and during hospital stay, and those prescribed at discharge, ADR, routine blood examination tests, cognitive function, admission and discharge diagnoses. All data were recorded at the clinical center on a microcomputer by means of a dedicated software. Such a software controlled the suitability and the internal consistency of the data so that impossible values or contradictory information could not be entered. The software allowed automatic coding of diagnoses, of ADRs and of drugs by simple typing the description of the disease, of the ADR, or of the commercial name of the drug. Procedures conformed to guidelines provided by the Catholic University Ethical Committee.

Overall, 17,186 patients were enrolled in the study period. Patients who died during hospital stay were excluded from the analysis to avoid the bias due to the presence of terminal illness. We selected five groups of patients on the basis of their first-listed diagnosis using the International Classification of Diseases 9^th ^revision Clinical Modification (ICD9-CM) codes [12]. Three groups consisted of patients with non-neoplastic chronic disease (congestive heart failure, N = 832; diabetes mellitus, N = 939; chronic obstructive pulmonary disease, N = 399), and were compared to three groups of patients with cancer: solid tumors (gastrointestinal, lung, breast, prostate, oro-pharyngeal, bone, and genito-urinary cancer) without metastasis (N = 813); solid tumors with metastasis (N = 259); leukemia/lymphoma (N = 326)

Variables specifically considered in this study were age, gender, length of hospital stay, number of diagnoses, use of drugs and prevalence of adverse drug reactions during hospital stay, and number of hospitalization in the last year. Functional capabilities were ascertained using the ADL scale [13], and categorical variables for dependency in at least 1 ADL or dependency in 3 or more ADLs were considered in the analysis. Cognitive status was evaluated by the Hodgkinson Abbreviated Mental Test (AMT), that is a 10-item version of the Blessed-Roth information-memory-concentration test [14,15], validated in an Italian population for screening for dementia [16]. Each question scores 1 point, and the total score ranges from 0 (no correct answer) to 10 (correct answers). On the weekday after admission, the study physician identified patients to be included in the study and interviewed them on the day before discharge to avoid any interference caused by an acute illness. The cut-off level of 7 (3 or more errors) has been reported to have 100% sensitivity and 71% specificity with respect to the DSM III diagnostic criteria of dementia [16].

We used contingency tables to compare the demographic and clinical characteristics of the groups studied. AMT and ADL scores of patients with lung or gastrointestinal cancer, i.e. of the most frequent cancers in the population studied, were separately analyzed to estimate the effects of metastases on the functional capabilities in homogeneous groups of cancer patients. Logistic regression analysis was used to obtain a deconfounded estimate of the association between the type of disease and physical or cognitive impairment. All analyses were performed using SPSS V10.0 (SPSS Inc., Chicago IL)[17].

## Results

The prevalence of patients aged 80 or more was higher in CHF and COPD than in diabetes and cancer groups, while male gender was more frequent in patients with COPD and cancer. Comorbidity was greater in diabetic patients, while patients with leukemia/lymphoma or metastatic cancer had the longest average stay. Use of NSAIDs and analgesics was greater in patients with diabetes and cancer, particularly in those with metastases. The highest rate of hospitalization in the previous year was observed in patients with COPD and cancer (Table 1).

**Table 1:** Demographic and clinical characteristics of the groups studied.

	CHF N = 842	Diabetes N = 939	COPD N = 399	Solid tumors N = 813	Leukemia/Lymphoma N = 326	Metastasis from solid tumors N = 259	P
Age, yrs							0.001
<65	8.4	29.9	16.3	20.9	27.3	30.1	
65–79	45.2	45.8	44.6	46.4	46.0	41.7	
80+	46.3	24.3	39.1	32.7	26.7	28.2	
Gender (males)	46.0	45.2	63.2	67.7	51.2	61.4	0.001
No of diagnoses>4	35.3	48.3	17.0	29.8	37.7	35.1	0.001
Length of stay>14 days	33.1	36.1	30.1	35.8	41.4	40.5	0.01
ADR during stay	12.1	10.4	6.3	7.4	7.7	10.4	0.002
Drugs during stay							
NSAIDs	3.6	10.5	4.8	13.7	15.6	28.6	0.001
Analgesics	0.2	8.0	1.3	3.8	3.1	6.9	0.001
More than 2 hospitalization in the last year	9.4	8.7	12.0	12.2	19.0	15.1	0.001

CHF = congestive heart failure; COPD = chronic obstructive pulmonary disease; ADR = adverse drug reactions; NSAIDs = non-steroidal antinflammatory drugs.

Data are percentage. P values in the last column refer to the 6 groups for 2 levels chi-square test.

**Figure 1:**
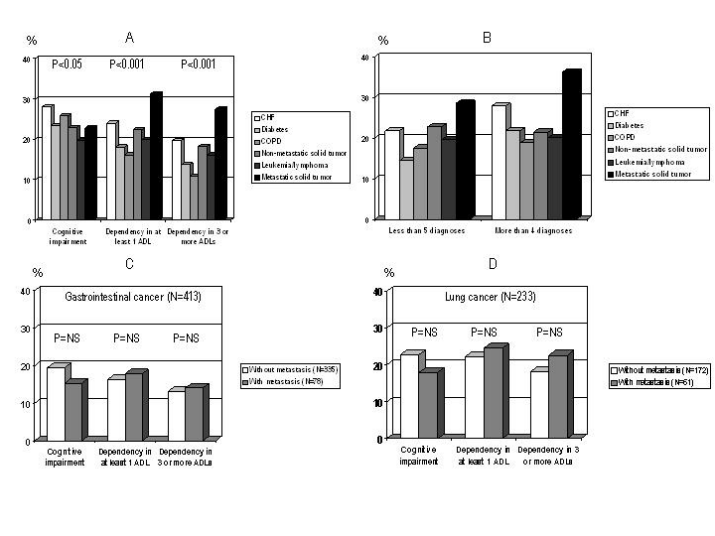
*Panel A: *prevalence of cognitive impairment and physical disability in patients divided according to their main diagnosis. *Panel B: *association between physical disability (dependency in at least 1 ADL) and comorbidity in the groups studied. *Panel C and D: *prevalence of cognitive impairment and physical disability in patients with gastrointestinal or lung cancer, with or without metastases.

Patients with cancer had a lower prevalence of cognitive impairment (22.9% in patients with solid tumors; 19.6% in patients with leukemia/lymphoma; 22.8% in patients with metastatic cancer) compared to patients with CHF (28.0%) or COPD (25.8%). Physical dependency was highly prevalent in patients with metastatic cancer (dependent in at least 1 ADL, 31.3%; dependent in 3 or more ADLs, 27.4%). The corresponding figures for patients with CHF were 24.0% and 19.6%, respectively (Figure 1, panel A).

Physical dependency in at least 1 ADL was significantly associated with higher comorbidity in patients with CHF (p < 0.05), diabetes (p < 0.01) or metastatic cancer (p < 0.05), but not in those with COPD, non-metastatic solid tumors or leukemia/lymphoma (figure 1, panel B). No significant association between cognitive performance and comorbidity was observed (data not shown).

For patients with gastrointestinal cancer (figure 1, panel C) and lung cancer (figure 1, panel D), the presence of metastases was associated to a slight increase in the prevalence of physical dysfunction, and to a slight decrease in the prevalence of cognitive impairment. However, these differences were not statistically significant.

**Figure 2:**
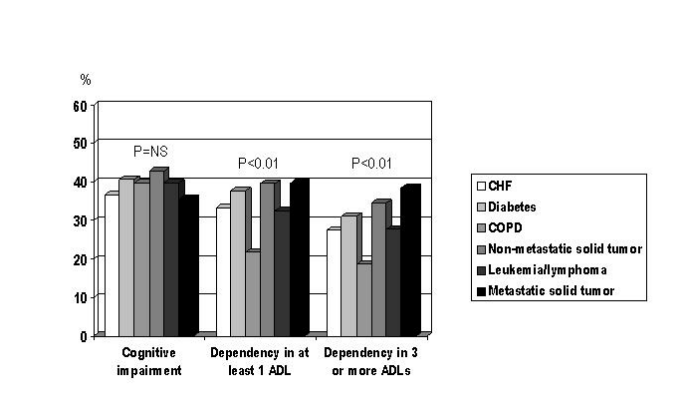
Prevalence of cognitive impairment and physical disability in patients aged 80 years or more (N = 1196) grouped according to their main diagnosis.

When we repeated the analysis in people aged 80 years or more (figure 2), we found that the prevalence of cognitive dysfunction was similar in all the conditions considered, including metastatic cancer. Furthermore, the prevalence of physical disability did not distinguish metastatic cancer from the remaining conditions.

**Figure 3:**
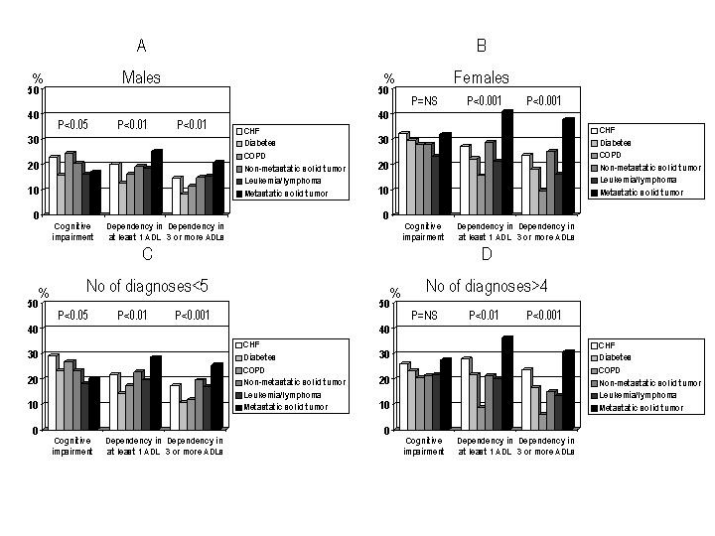
Prevalence of cognitive impairment and physical disability in: male patients divided according to their main diagnosis (*Panel A*); female patients divided according to their main diagnosis (*Panel B*); patients with less than 5 diagnoses divided according to their main diagnosis (*Panel C*); patients with more than 4 diagnoses divided according to their main diagnosis (*Panel D*).

The gender-specific limitations of functional capabilities are reported in figure 3. The highest prevalence of cognitive impairment was observed in males with CHF (23.0%) or COPD (24.6%) and in females with CHF (32.3%) or metastatic cancer (32.0%). Metastatic cancer was associated with the highest prevalence of physical dependency in both genders (Figure 3, Panel A and B). When we divided patients according to the number of diseases, CHF and COPD were associated with the greatest prevalence of cognitive impairment (29.2% and 26.9%, respectively) in patients with less than 5 diagnoses, while among patients with more than 4 diagnoses, the greatest prevalence of cognitive impairment was observed in CHF (25.9%) and metastatic cancer (27.5%) groups. Metastatic cancer was associated with the highest prevalence of physical dependency regardless of comorbidity (Figure 3, panel C and D).

Finally, after simultaneous adjustment for age, gender, number of drugs, number of diagnoses, and length of stay, only metastatic cancer was associated with a greater prevalence of physical but not cognitive impairment with respect to CHF patients. Diabetes was significantly associated with cognitive impairment (Table 2).

**Table 2:** Summary logistic regression models* of main diagnosis to cognitive impairment or physical dependency in at least 1 ADL.

	Cognitive impairment OR (95%CI)	Dependency in at least 1 ADL OR (95%CI)
CHF	1.0 (reference)	1.0 (reference)
Diabetes	1.40 (1.11–1.78)	0.93 (0.73–1.19)
COPD	1.13 (0.85–1.51)	0.73 (0.53–1.10)
Solid tumors	1.09 (0.85–1.39)	1.23 (0.95–1.57)
Leukemia/lymphoma	0.99 (0.70–1.38)	1.05 (0.75–1.47)
Metastatic cancer	1.34 (0.94–1.91)	2.09 (1.51–2.90)

*After adjusting for age, gender, number of drugs, number of diagnoses, and length of stay.

## Discussion

Our study indicates that physical performance and cognitive status in patients with non-metastatic cancer did not significantly differ from those observed in older hospitalized patients with other non-neoplastic chronic diseases. In presence of metastases, however, physical dependency was more severe, whereas cognitive impairment was significantly more prevalent in CHF than in metastatic cancer patients. Furthermore, compared to patients with CHF, those with metastatic cancer had longer hospital stay, greater number of hospitalization in the last year, and used more anti-inflammatory and analgesic drugs. Thus, our data confirm the common perception of metastatic cancer as a disease dramatically impacting on the health status, but this view should take into account the differential effect of cancer on physical and mental domains. On the other hand, non-metastatic cancer does not outweigh non-neoplastic chronic diseases as a cause of physical and cognitive impairment. It is interesting to note, however, that the presence of metastases had a distinctive impact on health status only in people aged less than 80. This finding may be consistent either with selective survival up to older ages or with a real lack of difference in the effects on health status of non-malignant chronic diseases (especially diabetes and CHF) and metastatic cancer in the very old.

Assessing the health status is relevant to optimize the therapy of cancer in the elderly. On average, the elderly are as likely to benefit from standard cancer treatment as younger people do [4]. Only older patients with functional and cognitive impairment are at higher risk of developing complications in response to aggressive treatments [18]. Thus, there is no sound basis for the common practice of treating the elderly with substandard therapy because of the perceived minimal benefit of chemotherapy and great risk of toxicity [18]. Age bias may affect both physicians' attitudes toward the use of standard anticancer therapeutic regimens in elderly patients [19], and the recruitment of elderly cancer patients in clinical trials [20,21]. Our data show that only one out of three older patients with metastatic cancer has severely impaired physical capabilities, whereas cognitive impairment is less common. Thus, in an unselected elderly population most of patients with metastatic cancer seem to be amenable to standard oncologic therapy, at least on the basis of their physical and cognitive capabilities.

The effect of CHF on health status is the object of a growing number of reports [22,23]. Our study adds to current knowledge by showing that CHF approaches metastatic cancer as a disabling condition, but, compared with metastatic cancer, it impacts more on mental than on physical capabilities. Indeed, cognitive dysfunction is highly prevalent in CHF populations and represents an important health problem, for example by affecting the compliance with therapy [22]. These findings might help understand the positive effects of geriatric assessment and intervention trial in CHF [24,25]. Indeed, physical rehabilitation and strategies enhancing the compliance with drugs and life style measures were a primary component of such trials [24]. Similarly, the association between diabetes and cognitive impairment is well known [26]. Our findings confirm this association and further stresses that metastatic cancer does not primarily affect mental performance.

Limitations of our study deserve to be cited. First, a cross-sectional observation is exploratory in nature, and it should be prospectively replicated. Furthermore, by considering only patients admitted to the acute care hospital, our sample can not be considered fully representative of the general population of older people. Second, the general health status of patients admitted to Geriatric or Internal Medicine units may be different from those admitted to Oncology or other specialty units. We excluded people dying during the hospital stay to avoid the bias introduced in the analysis by people with terminal illness. The GIFA questionnaire, however, does not contain an item on explicit terminal prognosis, therefore we could have excluded people with more advanced, but not terminal, disease. This could have biased our results by inflating the proportion of people with less advanced cancer progression. Nonetheless, by excluding people who died regardless of the diagnosis, we also excluded people with more advanced CHF or COPD, and this is likely to have offset the potential bias. Third, the use of a single cognitive screening test did not allow us to investigate the impact of selected chronic conditions on specific cognitive domains. Finally, comorbidity variously affects functional capabilities and thus might be responsible for some of the differences among groups. However, also in patients with more than 4 diagnoses, only the presence of metastatic cancer was associated with physical but not cognitive impairment. Furthermore, comorbidity usually characterizes patients having these main diseases, which makes present findings representative of the clinical reality.

## Conclusion

Cancer should not be considered as an ineluctable cause of severe cognitive and physical impairment, at least not more than other chronic conditions highly prevalent in older people, such as CHF. Further studies should be carried out to explain in which measure the impairment of mental and functional capabilities depends upon cancer per se or cancer related pain or comorbid conditions. Clarifying this issue in the individual patient would improve interventions aimed at reducing the burden of cognitive and physical dysfunction and improving health status in older patients with cancer.

## Competing interests

The author(s) declare that they have no competing interests.

## Authors' contributions

AC, CP and RAI participated in the conception and design of the study, analysis of data, and drafting of the manuscript. LC, FC and BM participated in the collection and analysis of data, and helped to draft the manuscript. All authors read and approved the final manuscript.

## Pre-publication history

The pre-publication history for this paper can be accessed here:


